# Enantioselective hydrolyzation and photolyzation of dufulin in water

**DOI:** 10.1186/1752-153X-7-86

**Published:** 2013-05-16

**Authors:** Kankan Zhang, Deyu Hu, Huijun Zhu, Jinchuan Yang, Jian Wu, Ming He, Linhong Jin, Song Yang, Baoan Song

**Affiliations:** 1 State Key Laboratory Breeding Base of Green Pesticide and Agricultural Bioengineering, Key Laboratory of Green Pesticide and Agricultural Bioengineering, Ministry of Education, Guizhou University, Guiyang 550025, China; 2 Research and Development Center for Fine Chemicals, Guizhou University, Guiyang 550025, China

## Abstract

**Background:**

Dufulin is a novel, highly effective antiviral agent that activatives systemic acquired resistance of plants. This compound is widely used in China to prevent and control viral diseases in tobacco, vegetable and rice. Dufulin can treat plants infected by the tobacco mosaic virus and the cucumber mosaic virus. However, the achiral analysis and residue determination of dufulin remain underdeveloped because of its high enantioselectivity rates and high control costs. The enantioselectivity of an antiviral compound is an important factor that should be considered when studying the effect of chiral pesticides on the environment. The enantioselective degradation of dufulin in water remains an important objective in pesticide science.

**Results:**

The configuration of dufulin enantiomers was determined in this study based on its circular dichroism spectra. The *S*-(+)-dufulin and *R*-(−)-dufulin enantiomers were separated and identified using an amylose tris-(3,5-dimethylphenylcarbamate) chiral column by normal phase high-performance liquid chromatography. The degradation of the *rac*-dufulin racemate and its separate enantiomers complied with first-order reaction kinetics and demonstrated acceptable linearity. The enantioselective photolysis of *rac*-dufulin allowed for the faster degradation of *R*-(−)-dufulin, as compared with *S*-(+)-dufulin. However, *S*-(+)-dufulin was hydrolyzed faster than its antipode.

**Conclusion:**

The photolysation and hydrolyzation of dufulin in water samples normally complied with the first-order kinetics and demonstrated acceptable linearity (*R*^2^>0.66). A preferential photolysation of the *R*-(−)-enantiomer was observed in water samples. Moreover, the *S*-(+)-enantiomer was hydrolyzed faster than its antipode.

## Background

Amino phosphonic acids are effective and environment-friendly analogs of natural amino acids. *N*-(Phosphonomethyl)glycine, more commonly known as glyphosate, is a representative amino phosphonic acid pesticide; it was first introduced in 1960s but eventually became one of the most popular pesticides worldwide. Related structural features can be found on (dl)-homoalanine-4-yl-(methyl)phosphonic acid, which is known as glufosinate and used as another herbicide [1]. The use of glyphosate and glufosinate has steadily increased over several decades, thereby making both the most frequently used herbicides worldwide. The success of glyphosate and glufosinate is attributed to their effective control of herb growth and their relative environmental safety; this level of safety may be attributed to their ability to bind to soil colloids for degradation by soil microbes [2-4].

**Figure 1:**
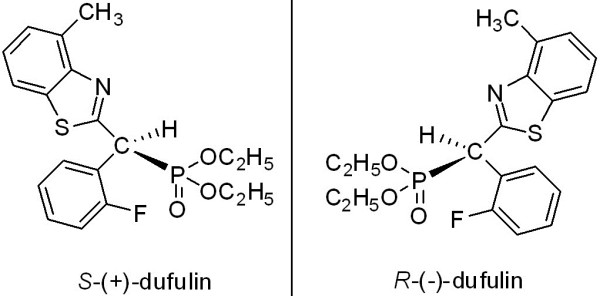
Chemical structures of the two enantiomers of dufulin.

Based on previous reports of amino phosphonic acids, our research team designed a series of novel heterocyclic α-amino phosphonic acid esters. Our group conducted an intensive effort to discover novel antiviral lead structures for plants, by optimizing α-amino phosphonic acids as lead compounds; a commercially registered plant antiviral agent named dufulin was eventually produced [5]. [(2-Fluoro-phenyl)-(4-methyl-benzothiazol-2-ylamino)-methyl]-phosphonic acid diethyl ester, also known as dufulin, has an asymmetric central carbon atom; this novel antiviral agent belongs to the α-amino phosphonate family (Figure 1). Dufulin is a highly effective against plant viruses; it functions by activating the systemic acquired resistance. This antiviral agent is used to control the tobacco mosaic virus (TMV) [6], the cucumber mosaic virus (CMV) [7], and Southern rice black-streaked dwarf virus (SRBSDV) [8] in China. A few methods for the achiral analysis and residue determination of dufulin have been reported in some matrices. However, to the best of our knowledge, the enantioselective degradation of dufulin in water has not been studied.

Current analytical methods for determining the presence of amino acid group-containing pesticides in water and other matrices are based on chromatographic techniques. The most commonly used chromatographic separation methods include high-performance liquid chromatography (HPLC) [9,10], gas chromatography (GC) [11], liquid chromatography-tandem mass spectrometry (LC/MS) [12], gas chromatography-tandem mass spectrometry (GC-MS/MS) [13,14], capillary electrophoresis (CE) [15-18], and enzyme-linked immunosorbent assay (ELISA) [2,3,19]. For example, an analytical method has been reported for quantifying dl-glufosinate enantiomers in biological specimens using precolumn derivatization and reversed-phase HPLC with a fluorescence detector. The lower limit of quantitation was 0.01 μg/mL for both l-glufosinate and d-glufosinate. The recovery rates of the two enantiomers from serum and urine were satisfactory [9].

A number of studies have shown that the toxicity of chiral amino acid pesticides is enantiomer specific. This finding suggests that the environmental behavior of pesticides containing amino acid groups should be studied using racemic compounds and their individual enantiomers [20-25]. For example, the absorption rate of d-glufosinate was lower than that of *rac*- or l-glufosinate in sugar beet [26]. In addition, the different biotransformations of *rac*-glufosinate, l-glufosinate, and d-glufosinate reportedly depend on the plant species. *rac*-Glufosinate and l-glufosinate were metabolized in non-transgenic and transgenic plant cell cultures, whereas d-glufosinate was not metabolized [27]. However, most chiral pesticides are released into the environment in their racemic forms as equimolar mixtures of enantiomers. Therefore, the enantioselective degradation of racemic amino acid pesticides is a major topic in pesticide degradation research [28].

In this paper, we investigated the enantioselective hydrolyzation and photolyzation of the racemate *rac*-dufulin and its individual enantiomers in water, as influenced by some environmental factors. A detailed knowledge of the kinetics, hydrolysis, and photolysis pathways of pesticides is pertinent during experimental design to obtain reliable rate constants for assessing the fate and transport of pesticide pollutants in aquatic ecosystems. Our results may have some implications on the environmental risk assessment of chiral pesticides.

## Results and discussion

### Absolute configuration of dufulin enantiomers

**Figure 2:**
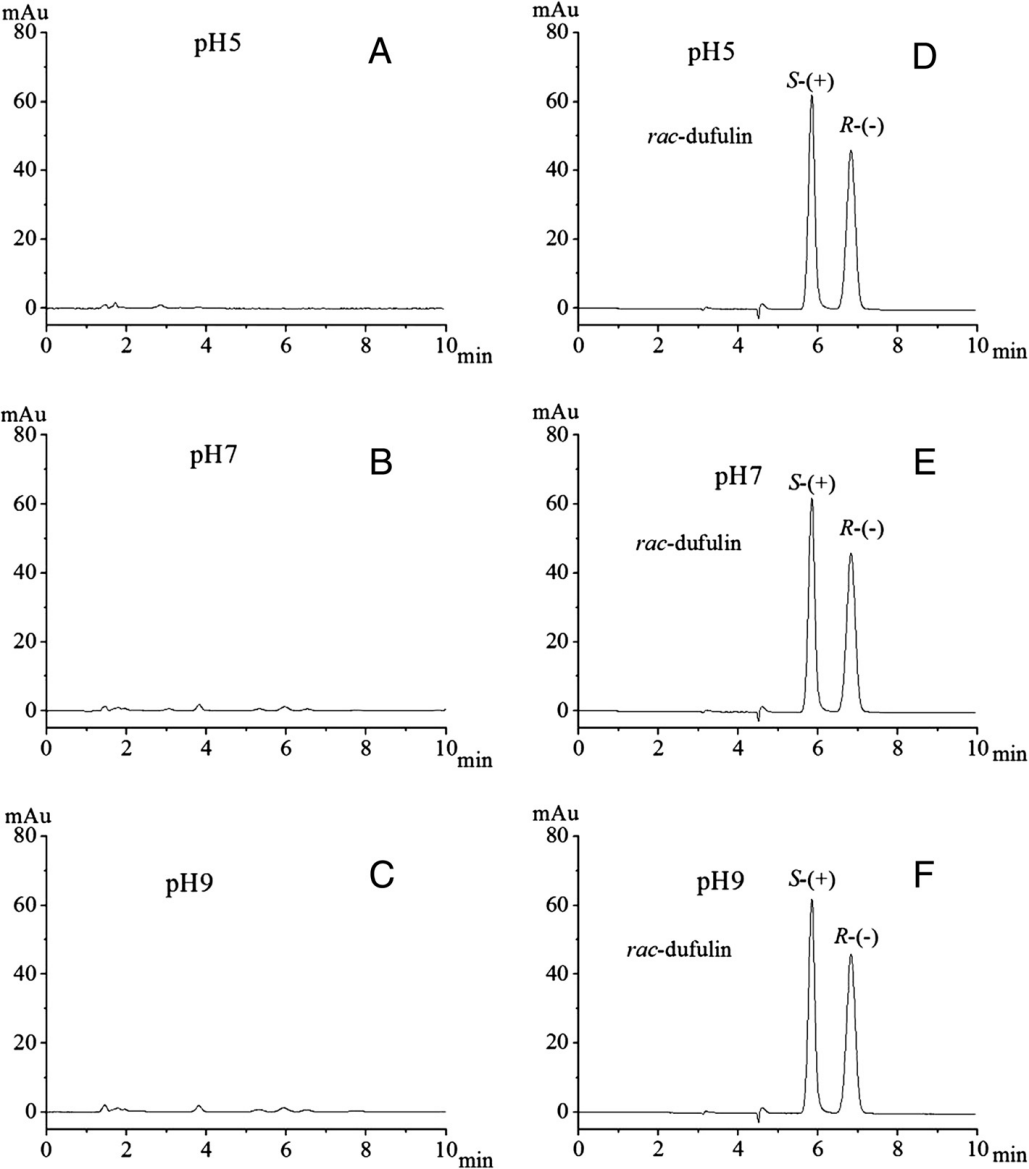
**HPLC chromatograms of blank and fortified water samples.** Blank water samples in different pH conditions: (**A**) pH=5; (**B**) pH=7; (**C**) pH=9; fortified water samples with *rac*-dufulin (spiked level 3.00 *μ*g/mL) in different pH conditions: (**D**) pH=5; (**E**) pH=7; (**F**) pH=9.

The individual enantiomers of dufulin were stereochemically analyzed by circular dichroism (CD) spectroscopy (NMR data of dufulin are shown in Additional file 1). Mirror-imaged CD curves were obtained by CD spectroscopy. The overall curves of the computed electronic circular dichroism (ECD) were obtained using time-dependent density functional theory (TDDFT) calculations. The computed and experimental ECD were similar to each other. The configurations of the dufulin enantiomers eluted from the columns could be correctly assigned by comparing the observed values with the absolute configurations of the computed ECD. The enantiomers represented by peaks 1 and 2 in Figure 2D were assigned as *S*-(+)-dufulin (which has better biological activity) [29] and *R*-(−)-dufulin, respectively.

### Calibration curves and assay validation

The calibration curves were plotted over the concentration range of 1.20 μg/mL to 60.0 μg/mL (*n* = 5) for each enantiomer in racemic dufulin. The regression equations and respective correlation coefficients were *y* = 12.549*x* + 2.5715 (*R*^2^ = 0.9998) for the *S*-(+)-enantiomer and *y* = 12.568*x* + 2.1867 (*R*^2^ = 0.9997) for the *R*-(−)-enantiomer. The recovery and precision data for the samples are summarized in Table 1. Each recovery value was acceptable for determining the enantiomers.

**Table 1:** **Precision, accuracy, and recovery data for the determination of *rac*-dufulin enantiomers (*n *= 5)**

**Enantiomer**	**Conc. (μg/mL)**	**ConcFound**^**a**^**(μg/mL)**	**Recovery**^**a**^**(%)**
*S*-(+)	0.30	0.28±0.05	92.01±16.77
*R*-(−)		0.27±0.04	90.00±13.33
*S*-(+)	1.50	1.55±0.09	106.3±1.56
*R*-(−)		1.56±0.09	104.0±6.00
*S*-(+)	3.00	3.19±0.05	103.7±5.74
*R*-(−)		3.20±0.04	106.6±1.36

^a^Values represent the means ± SD.

### Enantioselective photolyzation of dufulin in aqueous systems

**Figure 3:**
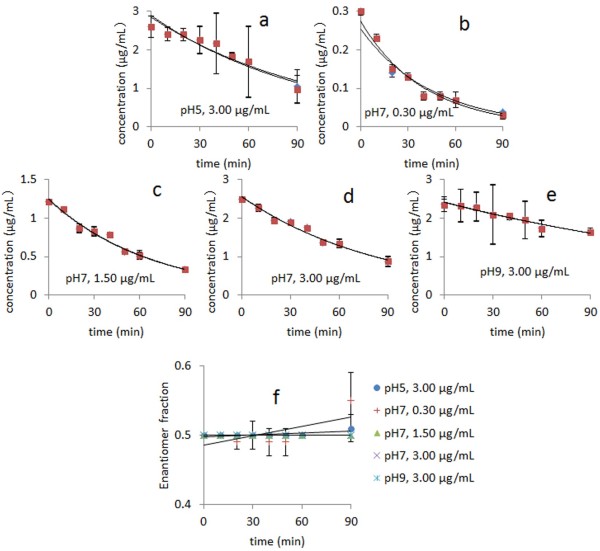
**Concentration-time and EF value-time curves of *rac*-dufulin photolyzed in water. ***rac*-Dufulin photolyzed in water samples under different conditions: (**A**) pH=5, spiked with 3.00 *μ*g/mL; (**B**) pH=7, spiked with 0.30 *μ*g/mL; (**C**) pH=7, spiked with 1.50 *μ*g/mL; (**D**) pH=7, spiked with 1.50 *μ*g/mL; (**E**) pH=9, spiked with 3.00 *μ*g/mL; (**F**) EF value-time curves. (♦ = *S*-(+)-enantiomer, ▄ = *R*-(−)-enantiomer. Values represent the means ± SD).

Water samples were spiked with either *rac*-dufulin or the individual enantiomers during the incubation period. The normal degradation of the dufulin enantiomers complied with first-order kinetics (see Tables 2 and 3, as well as Figure 3) while demonstrating acceptable linearity (correlation coefficient, *R*^2^ of 0.8891 to 0.9817). The photolyzation of dufulin enantiomers in water was influenced by the dufulin concentration and the reaction pH. The half-life (*t*_1/2_) values of the *S*-(+)-enantiomer in water at pH 7 were 31.4, 47.5, and 60.3 min for the *rac*-dufulin concentrations of 0.30, 1.50, and 3.00 μg/mL, respectively. By contrast, the *t*_1/2_ values of the *R*-(−)-enantiomer in water at pH 7 were 27.9, 47.5, and 60.8 min for the *rac*-dufulin concentrations of 0.30, 1.50, and 3.00 μg/mL, respectively. Therefore, the enantiomers were degraded faster as the concentration of dufulin was increased.

**Table 2:** **Photolytic regressive functions of *rac*-dufulin enantiomers in water (*n*=3)**

**Enantiomer**	**pH**	**Conc. (*μ*g/mL)**	**Regressive function**	**Parameters (*n*= 3)**		**ES value**^**a**^
				***R***^**2**^	***t***_**1/2**_**(min)**	
*S*-(+)	5	3.00	*C*_t_ = 2.8435 *e*^-0.0097*t*^	0.9181	71.5	0.030
*R*-(−)			*C*_t_ = 2.8860 *e*^-0.0103*t*^	0.8973	67.3	
*S*-(+)	7	0.30	*C*_t_ = 0.2534 *e*^-0.0221*t*^	0.9467	31.4	0.058
*R*-(−)			*C*_t_ = 0.2733 *e*^-0.0248*t*^	0.9741	27.9	
*S*-(+)	7	1.50	*C*_t_ = 1.2377 *e*^-0.0146*t*^	0.9797	47.5	0
*R*-(−)			*C*_t_ = 1.2477 *e*^-0.0146*t*^	0.9817	47.5	
*S*-(+)	7	3.00	*C*_t_ = 2.5561 *e*^-0.0115*t*^	0.9769	60.3	−0.0044
*R*-(−)			*C*_t_ = 2.5470 *e*^-0.0114*t*^	0.9808	60.8	
*S*-(+)	9	3.00	*C*_t_ = 2.4129 *e*^-0.0045*t*^	0.9522	154.0	0
*R*-(−)			*C*_t_ = 2.4014 *e*^-0.0045*t*^	0.9325	154.0	

^a^The ES value is defined as the enantiomeric selectivity value.

**Table 3:** **Photolytic regressive functions of *S*-(+)-dufulin and *R*-(−)-dufulin in water (*n*=3)**

**Compound**	**pH**	**Conc. (*μ*g/mL)**	**Regressive function**	**Parameters (*n*= 3)**	
				***R***^**2**^	***t***_**1/2**_**(min)**
*S*-(+)-dufulin	5	3.00	*C*_t_ = 2.6574 *e*^-0.0057*t*^	0.9599	121.6
*R*-(−)-dufulin			*C*_t_ = 2.8646 *e*^-0.0115*t*^	0.8891	60.3
*S*-(+)-dufulin	7	0.30	*C*_t_ = 0.2769 *e*^-0.0237*t*^	0.9685	29.2
*R*-(−)-dufulin			*C*_t_ = 0.2238 *e*^-0.0239*t*^	0.9624	29.0
*S*-(+)-dufulin	7	1.50	*C*_t_ = 1.4643 *e*^-0.0184*t*^	0.9712	37.7
*R*-(−)-dufulin			*C*_t_ = 1.4287 *e*^-0.0217*t*^	0.9810	31.9
*S*-(+)-dufulin	7	3.00	*C*_t_ = 2.5146 *e*^-0.0100*t*^	0.9769	69.3
*R*-(−)-dufulin			*C*_t_ = 2.7476 *e*^-0.0127*t*^	0.9808	54.6
*S*-(+)-dufulin	9	3.00	*C*_t_ = 2.5009 *e*^-0.0101*t*^	0.9522	68.6
*R*-(−)-dufulin			*C*_t_ = 2.3492 *e*^-0.0121*t*^	0.9325	57.3

The effect of pH is presented in Table 2. The *t*_1/2_ values of the *S*-(+)-enantiomer of *rac*-dufulin (3.00 μg/mL) in water were 60.3, 71.5, and 154.0 min at pH values of 7, 5, and 9, respectively. The *t*_1/2_ values of the *R*-(−)-enantiomer of *rac*-dufulin (3.00 μg/mL) in water were 60.8, 67.3, and 154.0 min at pH values of 7, 5, and 9, respectively. Dufulin photolyzation proceeded at higher rates under neutral conditions (with decreasing degradation rates at pH 7 > pH 5 > pH 9). Therefore, the photolytic process may be hindered in basic or acidic aqueous solutions. The photolysis of *rac*-dufulin was enantioselective in certain specific conditions, such as at pH 5 for 3.00 μg/mL or at pH 7 for 0.30 μg/mL, wherein the enantiomeric selectivity (ES) values were 0.030 and 0.058, respectively. The *R*-(−)-enantiomer was noticeably degraded faster than the *S*-(+)-enantiomer.

The preferential photolyzation of the single racemic dufulin enantiomer occurred at higher concentrations in neutral aqueous systems. The pure *R*-(−)-dufulin was photolyzed faster than its antipode under all different conditions (Table 3). The photolyzation rates of dufulin enantiomers in the racemate mixture were slower than those in the single-enantiomer form. This phenomenon might have been caused by the competition between the two enantiomers of *rac*-dufulin.

### Enantioselective hydrolyzation of dufulin in aqueous system

**Figure 4:**
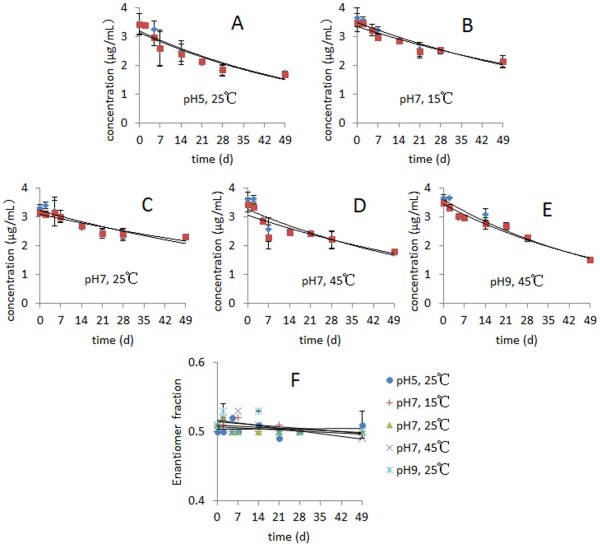
**Concentration-time and EF value-time curves of *rac*-dufulin hydrolyzed in water. ***rac*-Dufulin hydrolyzed in water samples under different conditions: (**A**) pH=5, 25°C; (**B**) pH=7, 15°C; (**C**) pH=7, 25°C; (**D**) pH=7, 45°C; (**E**) pH=9, 25°C; (**F**) EF values-time curves of *rac*-dufulin ( 3.00 μg/mL) in water. (♦ = *S*-(+)-enantiomer, ▄ = *R*-(−)-enantiomer. Values represent the means ± SD).

The hydrolytic degradation of dufulin enantiomers complied with first-order kinetics. The *R*^2^ values of 0.6659 to 0.9724 demonstrated acceptable linearity (Figure 4). The hydrolyzation of dufulin in water was strongly influenced by the pH, as shown in Tables 4 and 5. The *t*_1/2_ values of the *S*-(+)-enantiomer of *rac*-dufulin (3.00 μg/mL) in water at 25°C were 40.8, 46.2, and 61.9 d at pH values of 9, 5, and 7, respectively. By contrast, the *t*_1/2_ of the *R*-(−)-enantiomer of *rac*-dufulin (3.00 μg/mL) in water at 25°C were 43.6, 46.2, and 70.0 d at pH values of 9, 5, and 7, respectively. The *t*_1/2_ of dufulin demonstrated that dufulin hydrolysis proceeded at higher rates under alkaline and acidic conditions (with degradation rates of pH 9 > pH 5 > pH 7). These results implied that the reaction was more effectively catalyzed by hydroxide or hydronium ions than by the neutral water molecules. The incubation temperature similarly influenced the hydrolyzation of dufulin. The higher temperatures caused the faster hydrolysis of the dufulin enantiomers. The *S*-(+)-enantiomer, in particular, was degraded faster than its antipode in the experiments (except at pH 5). The ES values ranged from −0.099 to −0.033. These ES values likewise suggested that the hydrolysis of *rac*-dufulin was enantioselective. The *S*-(+)-enantiomer was degraded more rapidly than the *R*-(−)-enantiomer.

**Table 4:** **Hydrolytic regressive functions of *rac*-dufulin enantiomers (3.00 μg/mL) in water (*n*=3)**

**Enantiomer**	**pH**	**Temp. (°C)**	**Regressive function**	**Parameters (*n*= 3)**		**ES value**^**a**^
				***R***^**2**^	***t***_**1/2**_**(day)**	
*S*-(+)	5	25	*C*_t_ = 3.1994 *e*^-0.0150*t*^	0.8343	46.2	0
*R*-(−)			*C*_t_ = 3.1436 *e*^-0.0150*t*^	0.8739	46.2	
*S*-(+)	7	15	*C*_t_ = 3.2173 *e*^-0.0089*t*^	0.8174	77.9	−0.099
*R*-(−)			*C*_t_ = 3.0919 *e*^-0.0073*t*^	0.8429	95.0	
*S*-(+)	7	25	*C*_t_ = 3.4911 *e*^-0.0112*t*^	0.9425	61.9	−0.062
*R*-(−)			*C*_t_ = 3.3504 *e*^-0.0099*t*^	0.9211	70.0	
*S*-(+)	7	45	*C*_t_ = 3.2600 *e*^-0.0137*t*^	0.8574	50.6	−0.083
*R*-(−)			*C*_t_ = 3.0622 *e*^-0.0116*t*^	0.7813	59.8	
*S*-(+)	9	25	*C*_t_ = 3.5993 *e*^-0.0170*t*^	0.9502	40.8	−0.033
*R*-(−)			*C*_t_ = 3.4382 *e*^-0.0159*t*^	0.9724	43.6	

^a^The ES value is defined as the enantiomeric selectivity value.

**Table 5:** **Hydrolytic regressive functions of *S*-(+)-dufulin and *R*-(−)-dufulin (3.00 μg/mL) in water (*n*=3)**

**Compound**	**pH**	**Temp. (°C)**	**Regressive function**	**Parameters (*n*= 3)**	
				***R***^**2**^	***t***_**1/2**_**(day)**
*S*-(+)-dufulin	5	25	*C*_t_ = 3.1040 *e*^-0.0038*t*^	0.8448	182.4
*R*-(−)-dufulin			*C*_t_ = 3.1096 *e*^-0.0042*t*^	0.9705	165.0
*S*-(+)-dufulin	7	15	*C*_t_ = 3.0581 *e*^-0.0034*t*^	0.8943	203.9
*R*-(−)-dufulin			*C*_t_ = 3.0139 *e*^-0.0018*t*^	0.6853	385.1
*S*-(+)-dufulin	7	25	*C*_t_ = 3.1684 *e*^-0.0039*t*^	0.6659	177.7
*R*-(−)-dufulin			*C*_t_ = 2.9939 *e*^-0.0023*t*^	0.9204	301.4
*S*-(+)-dufulin	7	45	*C*_t_ = 3.0847 *e*^-0.0094*t*^	0.9833	73.7
*R*-(−)-dufulin			*C*_t_ = 2.8647 *e*^-0.0067*t*^	0.9078	103.5
*S*-(+)-dufulin	9	25	*C*_t_ = 3.0344 *e*^-0.0069*t*^	0.8720	100.5
*R*-(−)-dufulin			*C*_t_ = 2.7106 *e*^-0.0061*t*^	0.7780	113.6

Similar to *rac*-dufulin, the pure *S*-(+)-dufulin and *R*-(−)-dufulin compounds were hydrolyzed faster at higher temperatures in acidic or basic conditions. The hydrolyzation of *S*-(+)-dufulin was preferred (except at pH 5). However, the *t*_1/2_ values of the enantiomers in *rac*-dufulin were smaller than those of the individual enantiomers. This phenomenon could be attributed to the possible mutual promotion effects of the two enantiomers in racemic dufulin.

## Experimental

### Materials

*rac*-Dufulin (purity, >99%) was synthesized in pure form in our laboratory. The products were unequivocally characterized using a JEOL ECX 500 NMR spectrometer (JEOL, Tokyo, Japan). The spectrometer was operated at 500 and 125 MHz during ^1^H-NMR and ^13^C-NMR spectroscopy at room temperature. DMSO-d6 was used as the solvent, whereas tetramethylsilane was the internal standard. Elemental analysis was conducted using a Elementar Vario-III CHN analyzer (Elementar, Frankfurt, Germany). The pure enantiomers of dufulin were prepared using an Agilent HPLC system (Agilent, Wokingham, UK) with a semi-preparative chiral column. The purity of both enantiomers was greater than 99%. ECD spectroscopy was performed using a Jasco J810 spectropolarimeter (Jasco, Easton, USA) at room temperature. Water was purified using a Milli-Q system (Merck Millipore, Billerica, USA). The photolytic instrument was fabricated in-house. The radiant exposure, wavelength, and intensity of the ultraviolet lamp were set at 60 UW/cm^2^, 253.7 nm, and 20 lx, respectively. All other chemicals and solvents were of analytical-reagent or HPLC grade, as obtained from common commercial sources. The stock solutions of *rac*-dufulin and the two pure enantiomers were prepared in methanol and stored at −20°C. The water samples were obtained directly from a Milli-Q system. The aqueous solutions were prepared at different pH levels, and 0.1 mol/L NaOH was used to modify the pH of these solutions. The potassium acid phthalate solution (0.1 mol/L) was adjusted to pH 5, the potassium dihydrogen phosphate solution (0.1 mol/L) was adjusted to pH 7, and the boric acid/potassium chloride solution (0.1 mol/L) was adjusted to pH 9. The blank water samples showed undetectable levels of dufulin (Figures 2A, 2B, and 2C).

### Sample preparation

#### Photolyzation study

The water samples were spiked with dufulin by mixing the standard solution with methanol in 250 mL Quartz glass tubes and evaporating the solvent under a nitrogen flow (the experiments were conducted at pH 5, 7, and 9). Each tube was subsequently filled with 200 mL of each sample in water to produce the different concentrations of 0.30, 1.50, and 3.00 μg/mL. These samples were homogenized for 20 min by careful agitation, capped, and placed under the ultraviolet light (20 W). At exact sampling time schedule, a 20 mL sample was removed into the separatory funnel and immediately used for extraction.

#### Hydrolyzation study

The water samples were spiked with dufulin by mixing the standard solution with methanol in 500 mL Erlenmeyer flasks and evaporating the solvent under a nitrogen flow (the experiments were conducted at pH 5, 7, and 9). Each Erlenmeyer flask was subsequently filled with 200 mL of each sample in water to produce a nominal pesticide concentration of 3.00 μg/mL. The Erlenmeyer flask content was also agitated during 20 min, capped and placed at 25°C (or 15°C, or 45°C) in the dark. At exact sampling time schedule, 20 mL samples were removed into the separatory funnel and extracted immediately.

#### Sample purification

Each water sample were decanted into a 250 mL separatory funnel containing 20 mL distilled water and 10 mL 25% sodium chloride solution, and extracted with 30 mL, 20 mL dichloromethane, respectively. The lower layer was dried with anhydrous sodium sulfate, and then concentrated using a rotary evaporator (35°C, 0.07 MPa) and a nitrogen blow dry instrument (40°C). The residue was dissolved in 1.0 mL methanol, after filtration, an aliquot (5 μL) was injected into the HPLC for analysis. There was no detectable dufulin residue in blank water samples. All of the water incubation experiments were carried out in triplicate.

### ECD spectroscopy and chromatographic measurements

ECD spectroscopy was carried out by a Jasco-J810 spectropolarimeter at room temperature. The spectra were collected from 200 nm to 330 nm with a scan speed of 50 nm/min. The optimized geometry of two dufulin enantiomers was obtained by Gaussian 09 with density functional theory (DFT) at the B3LYP/6-31G* level. The ECD calculations of two enantiomers of dufulin were carried out by Gaussian 09 with TDDFT methods at the B3LYP/6-311++G (2d, 2p) level.

The analytical HPLC was performed using an Agilent 1200 Series apparatus equipped with a quaternary pump, an autosampler, a DAD detector (detection wavelength of 270 nm, reference wavelength of 360 nm, slit length of 4 nm), a vacuum degasser, a column oven and Agilent Chemstation software. After filtration, 5 μL of the sample was injected into the Chiralpak IA column (250 mm × 4.6 mm i.d., 5 μm, Daicel Chemical Industries Ltd.) with a guard column. The temperature of the column was adjusted to 25°C. The flow rate of the *n*-hexane–ethanol mobile phase (90:10, v/v) was 1 mL/min. The isocratic elution was used.

### Calibration curves and assay validation

For this study, a series of dufulin working standard solutions (1.20-60.0 *μ*g/mL) for linearity of the two enantiomers were prepared for HPLC analysis. Calibration curves were generated by plotting the peak area of each enantiomer against its concentration. Linear regression analysis was performed using Microsoft Excel 2010. The precision and accuracy of the method were calculated as the ratio of the spiked concentration of each enantiomer in blank treatment (with water alone) to the predicted concentration. The recoveries of enantiomers were determined by analyzing the quality control samples at three different fortified levels. The samples were extracted, and determined as previously described. The concentration of each enantiomer in water was calculated from calibration curves of the corresponding enantiomer using an external standard.

### Kinetic analysis and calculation

For the photolytic and hydrolytic treatments, the data were assumed followed first-order kinetics model, corresponding rate constant *k* for the (+)-enantiomer and (−)-enantiomer were calculated according to (1). The starting point was the maximum value of the concentration, and declined in following days. The half-life (*t*_1/2_ day) was determined from (2).

(1) $$C_{t}=C_{0}e^{-\mathrm{kt}}$$

(2) $$t_{1/2}=\ln2/k$$

The enantiomer fraction (EF) was used to express enantioselectivity as defined by equation: EF = concentration of the (+) / ((+) + (−)). The EF values range from 0 to 1, with EF = 0.5 representing the racemic mixture. The enantiomeric selectivity (ES) value was used to reflected the overall trend in enantioselective degradation process [30,31]. ES was defined by equation as follows: ES = (*k*_(−)_ - *k*_(+)_) / (*k*_(−)_ + *k*_(+)_). Positive values (0 < ES ≤ 1) indicate a more rapid degradation of (−)-enantiomer, while negative (−1 ≤ ES < 0) indicate a more rapid degradation of (+)-enantiomer. When ES value is 0, degradation is not enantioselective.

## Conclusion

In conclusion, the absolute configurations of dufulin enantiomers were determined by ECD spectra, the first eluted enantiomer was *S*-(+)-dufulin, the second one was *R*-(−)-dufulin. The extract and detection methods were optimized. The precision and accuracy data showed that these methods were satisfied with the experiments. The photolysation and hydrolyzation of *rac*-dufulin in water samples normally complied with the first-order kinetics, with acceptable linearity (*R*^2^>0.66). The preferential photolyzation of *R*-(−)-dufulin occurred in certain water samples. Moreover, *S*-(+)-dufulin was hydrolyzed faster than its antipode. Future investigations should focus on the mechanisms of enantioselective sorption–desorption and the mobility behavior of dufulin in the soil, which may provide insights for improved environmental and ecological risk assessment.

## Competing interests

The authors declare that they have no competing interests.

## Authors’ contributions

The current study is a product of constructive discussions among BAS, LHJ, DYH, and SY, who offered necessary guidance to KKZ, HJZ, JCY, and MH while conducting their experiments. KKZ was likewise involved in drafting the manuscript. KKZ, HJZ, and JCY performed the data analysis. BAS and JW were involved in revising the manuscript. All authors have read and approved the final manuscript.

## Supplementary Material

Additional file 1 The physical and NMR spectral data of dufulin. Physical property, melting point and ^1^H-NMR and ^13^C-NMR data of dufulin. Click here for file
