# Supplementary material for: Enantioselective hydrolyzation and photolyzation of dufulin in water
Source: Chem Cent J. 2013 May 16;7:86. doi: 10.1186/1752-153X-7-86 (PMC3663813; doi:10.1186/1752-153X-7-86)
Supplement: Additional file 1 — The physical and NMR spectral data of dufulin. Physical property, melting point and 1H-NMR and 13C-NMR data of dufulin. [file 1752-153X-7-86-S1.doc]

Additional file 1

**Enantioselective hydrolyzation and photolysation of dufulin in water**

Kankan Zhang, Deyu Hu, Huijun Zhu, Jinchuan Yang, Jian Wu Song Yang , Baoan Song*

Address: State Key Laboratory Breeding Base of Green Pesticide and Agricultural Bioengineering, Key Laboratory of Green Pesticide and Agricultural Bioengineering, Ministry of Education, Research and Development Center for Fine Chemicals, Guizhou University, Guiyang 550025, China.

**The physical and NMR spectral data of dufulin are listed below.**

**[(2-Fluoro-phenyl)-(4-methyl-benzothiazol-2-ylamino)-mehyl]-phosphonic acid diethyl ester (dufulin).**

**
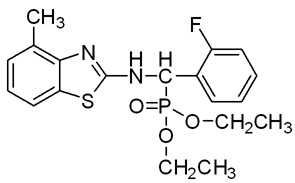
**

White solid, M.p. 143-145 °C. ^1^H-NMR (500 MHz, CD_3_Cl) δ: 7.97 (t, *J* = 7.2 Hz, 1H, benzene H), 7.75 (t, 1H, *J* = 7.2 Hz, benzene H), 7.24 (t, 1H, *J* = 7.3 Hz, benzene H), 7.10-7.14 (m, 3H, benzene H), 6.94 (t, 1H, *J* = 7.7 Hz, benzene H), 6.05 (dd, 1H, *J* = 11.2 Hz, NH), 4.29 (m, 2H, CH_2_), 4.05 (m, 1H, CH), 3.86 (m, 1H, CH), 2.52 (s, 3H, CH_3_), 1.32 (t, 3H, *J* = 7.2 Hz, CH_3_), 1.14 (t, 3H, *J* = 7.2 Hz, CH_3_); ^13^C-NMR (125 MHz, DMSO-*d_6_*): δ 164.66, 161.79, 131.16, 129.69, 129.37, 126.49, 124.49, 123.46, 121.71, 118.17, 115.37, 63.88, 49.35, 48.11, 18.29, 16.58, 16.31.
